# Attenuated *P. falciparum* Parasite Shows Cytokine Variations in Humanized Mice

**DOI:** 10.3389/fimmu.2020.01801

**Published:** 2020-09-11

**Authors:** Lei-lei Zhang, Jin-Long Li, Ming-Xin Ji, Dan Tian, Li-Yan Wang, Chen Chen, Miao Tian

**Affiliations:** ^1^Department of Anesthesiology, The Second Hospital of Jilin University, Changchun, China; ^2^Department of Gastrointestinal Surgery, The Second Hospital of Jilin University, Changchun, China; ^3^Department of Operating Room, The Second Hospital of Jilin University, Changchun, China; ^4^Department of Gynecology and Obstetrics, The Second Hospital of Jilin University, Changchun, China

**Keywords:** humanized mice, clodronate-loaded liposomes, NOD/SCID/IL-2rg^−^/ (NSG) growth mutants, TK/NOG, cytokine, PF3D7_1305500, C9 parasite mutants (C9-M), complemented parasites (C9-C)

## Abstract

A recently developed humanized mouse has been used to assess the immune response evoked against the isolated attenuated C9 parasite clone (C9-M; carrying a single insertion disrupting the open reading frame (ORF) of PF3D7_1305500) of *Plasmodium falciparum*. Significant human RBC engraftment was achieved by ameliorating the residual non-adaptive immune response using clodronate-loaded liposome treatment. Controlled reactive professional phagocytic leukocytes in immunodeficient mice allowed for sizeable human blood chimerism and injected huRBCs acted as *bona fide* host cells for *P. falciparum*. huRBC-reconstituted immunodeficient mice received infectious challenge with attenuated *P. falciparum* C9 parasite mutants (C9-M), complemented (C9-C), and wild type (NF54) progenitors to study the role of immune effectors in the clearance of the parasite from mouse circulation. C9-M and NF54 parasites grew and developed in the huRBC-reconstituted humanized NSG mice. Further, the presence of mutant parasites in deep-seated tissues suggests the escape of parasites from the host's immune responses and thus extended the survival of the parasite. Our results suggest an evasion mechanism that may have been employed by the parasite to survive the mouse's residual non-adaptive immune responses. Collectively, our data suggest that huRBCs reconstituted NSG mice infected with attenuated *P*. *falciparum* is a valuable tool to explore the role of C9 mutation in the growth and survival of parasite mutants and their response to the host's immune responses. This mouse might help in identifying novel chemotherapeutic targets to develop new anti-malarial drugs.

## Introduction

The human malaria parasite was accountable for 4,45,000 deaths in the 2016 (1). The *in vitro* findings do not replicate the *in vivo* findings and therefore a laboratory animal model is indeed needed. However, the study of human malaria parasites in animal models is severely limited by ethical and technical constraints, since only a few primate species have been found to be receptive to *P. falciparum* infection (2–4). Currently, the majority of *in vivo* investigations to understand malaria biology are dependent upon rodent malaria species (*P. berghei* and *P. yoelii*) which are used as surrogates to study human malaria (3–6). Therefore, humanized mice capable of harboring the human malaria infection are urgently needed to understand the parasite biology. A human blood chimeric mouse could serve to harmonize *in vitro P. falciparum* cultivation and *in vivo* studies carried out in rodent animal models. Introduction of several mouse strains with genetic immune deficiencies has greatly benefited the development of a small laboratory animal model (7–15) to study the asexual blood stage infection of *P. falciparum*. Recently, an immunodeficient mouse (16) was reconstituted with uninfected and infected huRBCs. This NSG mouse, depleted with γ-chain of the IL-2 receptor, has been shown to better tolerate a variety of human transplanted cells (17–24). The reduction in the residual innate immune effectors (mainly cells of monocytes and macrophages lineage) and co-administration of huRBCs supplied with decomplemented human serum through an intravenous route led to the development of a reproducible humanized mouse. The disruption of PF3D7_1305500 in C9-M parasites showed 50% attenuation as compared to the wild type parasites (NF54) (25). Therefore, C9-M and C9-C (Rescued phenotype of wild-type growth by genetic complementation) parasites (26) showed the attenuation in PF13_0027 knock-out parasites (C9-M) growth, which in turn resulted in the irregular cell cycle. The late entry into the S/M phase coincides with the timing for the peak expression of PF13_0027, suggesting that the deficiency in the mutant cycle can be correlated with the gene expression pattern (26).

Low parasitemia of the C9-M parasite in mouse circulation and extended survival of the C9-M parasite in deep-seated tissues suggests that the parasite may have employed a mechanism to escape the host's residual immunity. This parasitologically altered behavior of C9-M parasites was confirmed by the serum estimation of pro-inflammatory cytokines from the C9-M infected mice when compared to the NF54 and C9-C parasites.

Thus, the present study was designed to create an improved understanding of host–parasite interactions to bridge the gap between *in vivo* and clinical studies. The growth mutant (C9-M) parasites grafted in humanized mice showed nearly similar parasitemia patterns to that of NF54. Further, the C9-M parasite seems to have employed a mechanism to evade the host's immune responses and resides/sequesters in the deep-seated tissues. Our study showed the importance of human RBC reconstituted NSG mouse model to study the behavior of generated attenuated and complement parasites (25, 27). This humanized mouse may prove to be an important tool to study the immune mechanism(s) employed by the parasite to develop an indepth understanding on the sequestration-like phenomenon of human malaria parasite, *P. falciparum*. In brief, the present study showed the variation in the cytokines and parasitologically altered behavior of C9-M parasite upon engrafted in huRBC reconstituted NSG mice for its extended survival.

## Materials and Methods

### Animal Ethics Committee Approval

All animal procedures were carried out in compliance with The Second Hospital of Jilin University, Changchun. The procedures were reviewed and approved by The Second Hospital of Jilin University, Changchun, China.

### Mice

Four- to 6-week-old male and female NOD/SCIDIL-2Rγ^−/−^ (NSG/NOG) mice were procured from Jackson Laboratory, USA. The transgenic thymidine kinase-NOG (TK/NOG) strain was procured from Taconic, USA. The immunodeficient/transgenic mice were housed in sterile isolators and supplied with autoclaved tap water with a γ-irradiated pelleted diet *ad libitum*. Animals were manipulated under pathogen free conditions using a laminar flow-hood cabinet. One hundred and ten animals were used in the study. Six animals were allocated for each experimental group, except for the giemsa staining assay wherein four animals were used. Animal allocation was randomized, and mice receiving treatment were monitored daily and weighed three times (Monday, Wednesday, Fridays) per week. *P. falciparum* infected mice were treated with the analgesic ketoprofen to alleviate pain caused by the malaria infection.

### Human Erythrocytes

The huRBCs are the *bona fide* host cells for the development of *P. falciparum* in *in vitro* cultivation and huRBCs reconstituted mice. The packed huRBCs were provided by the Interstate Blood Bank (Chinese blood bank). Blood was taken from donors with no history of malaria. The huRBCs were suspended in SAGM (Saline, Adenine, Glucose, Mannitol solution) and stored at 4°C for a maximum of 2 weeks. Before injection, huRBCs were washed three times in RPMI-1640 medium (Gibco-BRL, Grand Island, New York), supplemented with 1 mg of hypoxanthine per liter (Sigma-Aldrich, St. Louis, Missouri), and warmed for 10 min at 37°C. Blood samples drawn from mice were used to determine the percentage of huRBC in mice's peripheral blood at regular intervals by flow cytometer (Accuri cytometers) using FITC labeled anti-human glycophorin antibody (ebiosciences, USA).

### *P. falciparum* Parasites Culture

C9 mutant, complement parasites (Gene complementation of the C9 mutant to rescue the wild-type phenotype) and wild type *P. falciparum* line (NF54) were employed in the study. C9-M and C9-C parasites were generated by the interstional mutagenesis by others (26). *P. falciparum* strains were cryopreserved using the glycerol/sorbitol method as described elsewhere (28). The parasites were cultured *in vitro* at 5% hematocrit, at 37°C with 5% CO_2_, using RPMI-1640 medium (Gibco/BRL), 35 mM HEPES (Sigma), 0.25% NaHCO_3_, 0.5% albumax II (Gibco/BRL), and 0.01 mg/ml gentamicin.

### *In vivo* Replication of *P. falciparum* in the NSG-IV Model

*P. falciparum* was maintained in huRBCs reconstituted NSG (immunocompromised) mice undergoing additional modulation of innate immune-defenses using the clodronate-containing liposomes, as described earlier (16). huRBC administered and intravenously infected humanized mouse is referred to “NSG-IV model.” The proportion of huRBCs in the mouse's blood was measured at 3-day intervals until the end of the study by the flow cytometer (Accuri C6 flow cytometry, BD Biosciences, USA) using FITC labeled anti-human glycophorin monoclonal antibody (ebiosciences, CA, USA). Seventy to Ninety percentage of circulating huRBCs were quantified in the mice circulation. Mice were intravenously inoculated with 300 μl asynchronous *P. falciparum* culture maintaining 1% parasitemia. Following *P. falciparum* infection, thin blood films were drawn daily from the tail vein on infected humanized mice. Parasitemia has been expressed as a percentage of all erythrocytes found in mouse periphery; the real percentage of parasitized huRBCs is higher in humanized mice, proportional to the level of chimerism, since murine erythrocytes do not receive infection but were included in counts.

Estimates of the total parasite biomass in each mouse were calculated based on the mean corpuscular volume (MCV) of mouse erythrocytes (45fL), MCV of human erythrocytes (86fL), hematocrit in the mice of 0.7, weight of NSG mice (25 grams), and a conservative estimate of 5.5 ml of blood per 100 grams of mouse weight using the following equation:

Number of infected RBCs = (0.055 ml/g) (25 g) (0.7) × (huRBC parasitemia)

[86fL + (mouse Chimerism/human Chimerism) 45fL]

### Sorbitol Synchronization of *Plasmodium falciparum*

Parasite cultures at 5% parasitemia, predominantly ring stages, were synchronized to remove late stage parasites using pre-warmed (37°C) 5% sorbitol. After harvesting culture at 1600 RPM for 6 min, medium was removed and replaced with 10 volumes of pre-warmed 5% sorbitol (29). The re-suspended culture was incubated at 37°C for 15 min, and parasites were pelleted down by the centrifugation; sorbitol was then removed and replaced with fresh complete medium.

### Giemsa Staining and Parasite Count

*P. falciparum* infected mice were euthanized to extract organs (Kidney, liver, spleen, lung, and brain). These extracted organs were perfused, and cells collected from the organs were placed on the glass slides. Briefly, cells were fixed with methanol and stained with Giemsa, and examined at 100X magnification to perform differential counts of each stage (200 parasites from each organ counted). The analysis of deep-seated organs for parasite count in NSG mice infected with C9-M and C9-C parasites was carried out.

### Genotyping of Growth Attenuated (Mutant) and Complemented *P. falciparum* Parasites

The original parasites C9-M and C9-C were harvested from *in vitro* cultures as well as from humanized mouse (huRBCNSG-IV) infected with C9-M, C9-C, and wild type parasites. The specific primers (F5′ATGGTTGGTTCGCTAAACTG3′, R5′TTAATCATTCTTCTCATATACTTCAAA3′) and (F:CTTCACTATCGCTTTGATCC, RTCGCTATCCCATAAATTACAA) were used to identify the presence of hDHFR and BSD from *in vitro* cultures and *P. falciparum*-harboring humanized mice. The genomic DNA was extracted from parasite cultures and tail snips of mice infected with *P. falciparum* strains using a DNA mini-kit (Qiagen). DNA was amplified in 20 μl reaction mixture by adopting the following PCR conditions: 1 cycle at 45°C for 30 min and 94°C for 2 min, followed by 35 cycles of 94°C for 15 s, 45°C for 30 s, and 65°C for 3 min. The amplified DNA showed the product band of 563 and 393 bp on 0.8% agarose gel of hDHFR and BSD, respectively.

### *Plasmodium falciparum* Growth Assay

Growth assays were carried out by maintaining asynchronous cultures of *P. falciparum* wild-type and mutant parasites at 0.5–2% parasitemia in 96-well plates and diluting every 48 h for 168 h. The parasite cultures were plated in triplicate at time zero and end point of the assay, and for each time point samples were taken at every 24 h for 7 days and fixed with 0.05% glutaraldehyde after the removal of culture medium. Flow cytometer was used to estimate the parasitemia as described elsewhere (30, 31) and parasites were stained with ethidium bromide. The stained parasites were analyzed through Accuri C6 flow cytometry system (Accuri, USA). A total of 100,000 cells were counted for each sample and data were analyzed using C Flow Plus software (Accuri). Growth rate (defined as the change in parasite numbers every 24 h over a period of 7 days) analyses were performed using Microsoft Excel and (Microsoft) and SAS 9.3.

### Serum Estimation of Cytokines and Chemokines

Hundred microliter blood samples were collected through the submandibular puncture of mice, and sera were stored at−80°C. Cytokines and chemokines (IL-6, MCP-1, IFNγ, TNFα, IL-12p70, and IL-10) were quantified using the BD^TM^ Cytometric Bead Array mouse inflammatory kit (BD biosciences) following the manufacturer's recommendations.

### Genotyping of TK/NOG Mice by Diagnostic PCR

Transgenic offspring were genotyped and identified by PCR (annealing temperature 59°C) using HTKF1 forward primer, 5′-CACGTCTTTATCCTGGATTACG-3′ and hGHR1 reverse primer, 5′-CACTGGAGTGGCAACTTCCA-3′. The genomic DNA was extracted from tail snips by DNA mini-kit (Qiagen), and amplified in a 20 μl reaction mixture using the PCR conditions: 2 min at 94°C, then 30 cycles of 30 s at 94°C, 30 s at 59°C, and 30 s at 72°C, and finally 3 min at 72°C. The transgene DNA showed an amplified product band of 236 bp on 1% agarose gel (32).

### Statistical Analysis

Each growth assay data was analyzed using C Flow Plus software (Accuri). Growth rate and statistical analysis was carried out by Student's *t*-test using Prism software (Graph Pad 5 Demo) and data was expressed as the mean ± standard deviation (S.D.) of the mean (^*^p < 0.05, ^**^p < 0.01, and ^***^p < 0.001). A value of *P* < 0.05 was considered statistically significant. The evaluation of inflammatory mediators' results are presented as mean SEM from the experiment performed using three mice per group.

## Results

### Growth and Replication of *P. falciparum* in PfhuRBC-NSG-IV Mice

**Figure 1:**
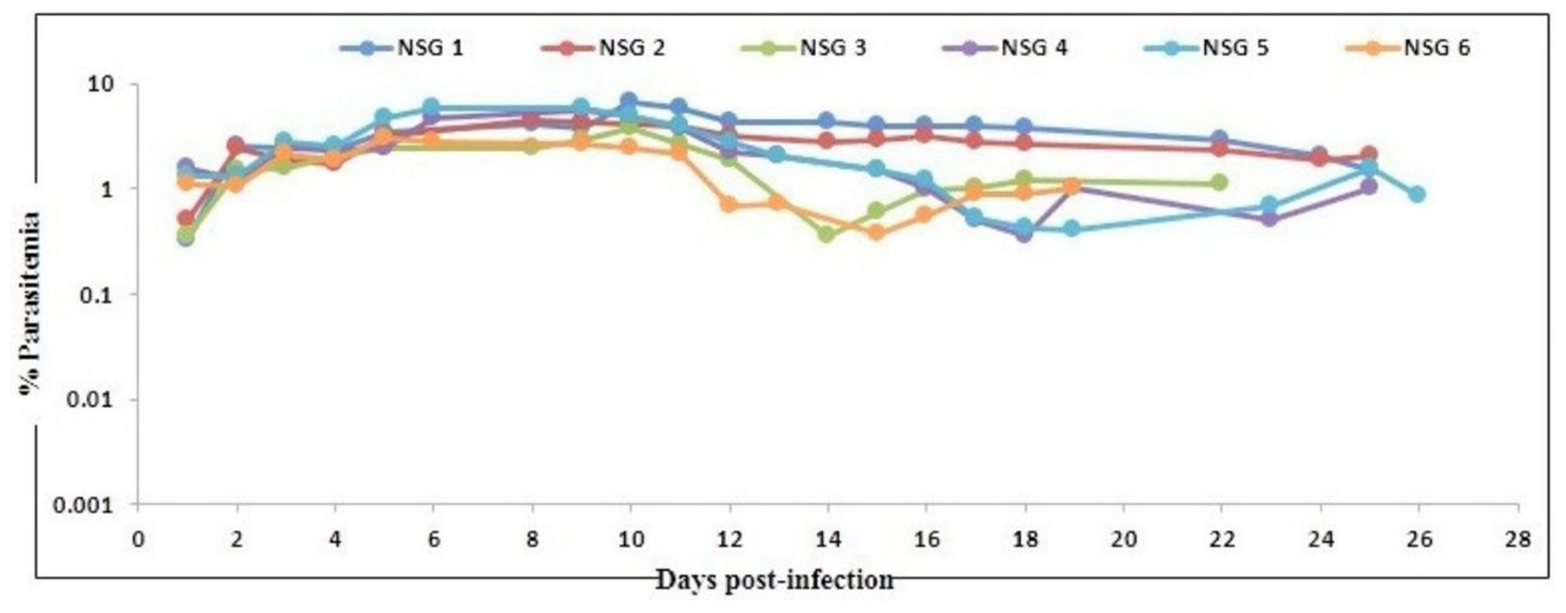
Evolution of *P. falciparum* (NF54) parasitemia in six untreated mice. Mice were intravenously inoculated with 0.3 ml of asynchronous cultures at 1% parasitemia of *P. falciparum* on day 0. Mice were supplied with huRBC every 3 days. Parasitemia in mice was expressed as the percentage of *P. falciparum-*huRBC in the total RBC observed on thin blood smears. Data shown are from the first day of detectable parasitemia up to the day the mice were used for other malaria studies.

**Figure 2:**
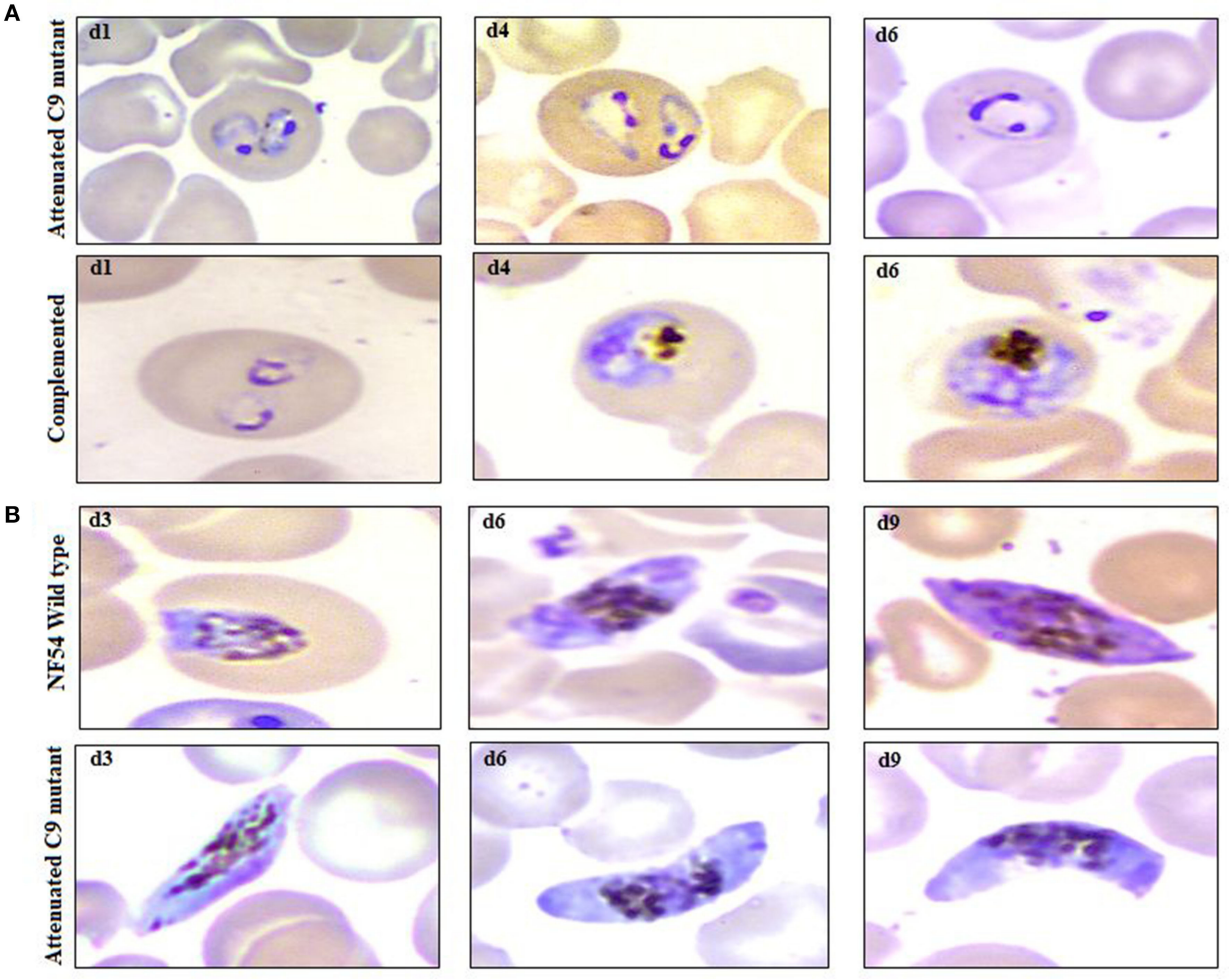
Example of parasitemia obtained from highly attenuated C9-M and complemented C9-C parasites in humanized mice (NSG-IV), **(A)** upper row (attenuated C9 mutant) and lower row (complemented) depicts the dominance of ring stage parasites along with the presence of trophozoites with frequent poly-parasitism seen on thin blood smears drawn from NSG mice infected with C9-M and C9-Cparasites, respectively, on day 1, 4, and 6 post-infection, **(B)** occurrence of mature gametocytes (stage V) in peripheral blood of NSG mice infected with NF54 (upper row) and attenuated C9 mutant (lower row) *P. falciparum*.

In agreement with the previous findings (16), we used a slightly modified protocol for reconstituting NSG immunodeficient mice with huRBCs (PfhuRBC-NSG) for sustained *P. falciparum* growth. Experiments were performed in NSG mice using 650 μl of huRBC pellets mixed with 25% de-complemented human serum intravenously injected three times every week, and reconstituted NSG mice were infected with *P. falciparum* through an intravenous route. Codronate-loaded liposome (clo-lip) suspension was injected intraperitoneally, and clo-lip and huRBCs were administered the same day. This mouse model is called “Pf-NSG IV” model. With this IV protocol 100% of NSG mice, as reported earlier (16), were seen parasitized by day 1 post-inoculation. As shown in Figure 1, low to moderate levels of parasitemia were obtained. In fact, parasites could persist as long as un-infected huRBC were co-administered with human serum along with the immunomodulatory agent, clo-lip. Furthermore, despite individual variations in the maximal parasitemia reached, the parasitemia observed were found stable to determine *in vivo* effect of C9-M *P. falciparum* parasites. Thus, we sought to better understand the individual variations in the parasitemia in mouse circulation which could be explained due to the varying levels of blood chimerism. However, significant (*p* < 0.05) numbers of huRBCs in mouse periphery supported rapid and optimum growth of *P. falciparum* (Figure 1) with abundant and healthy-looking parasites showing frequent poly-parasitism of huRBC (Figure 2A).

**Figure 3:**
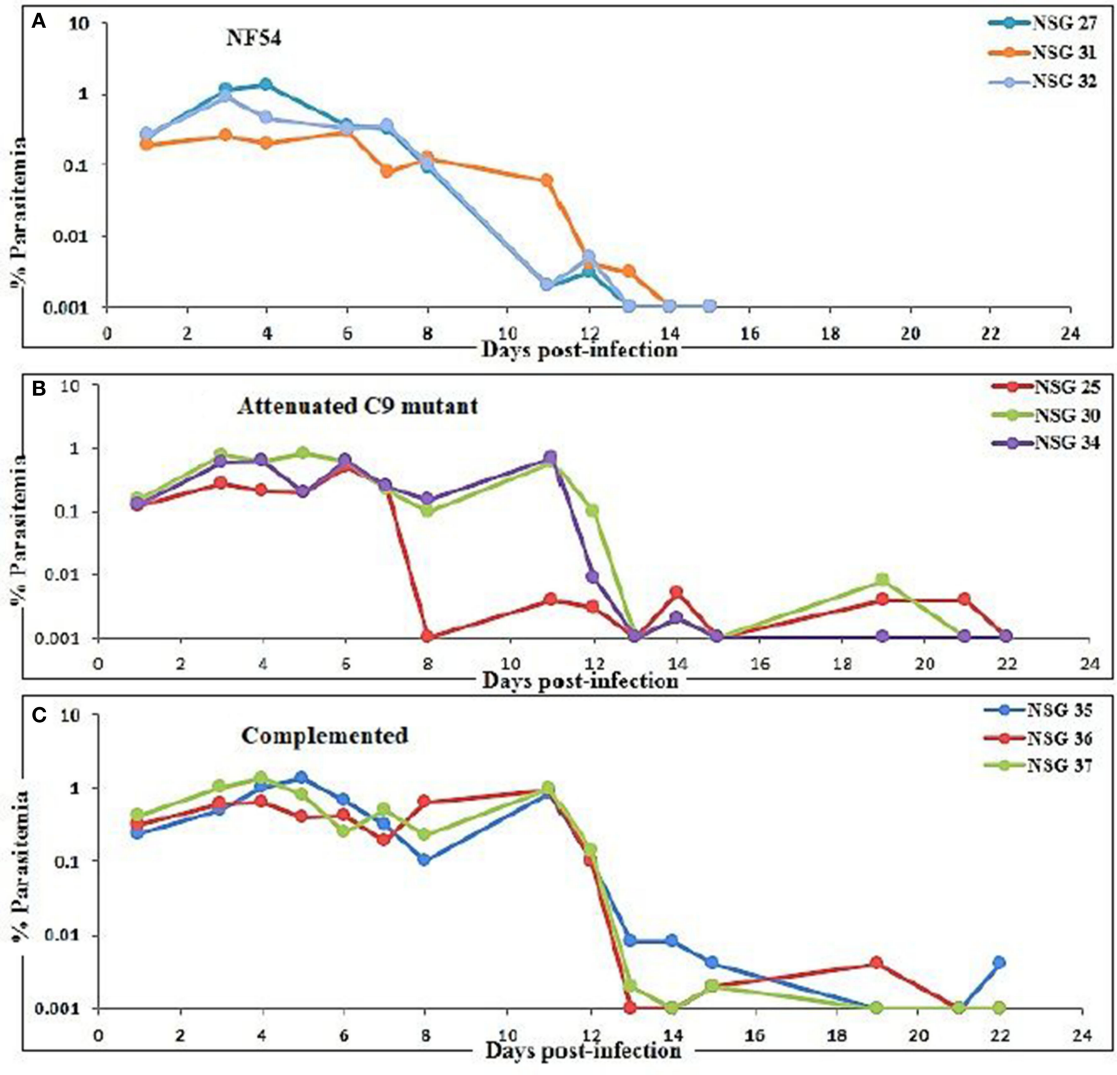
Evolution of *P. falciparum* parasitemia of mice treated with **(A)** NF54 (wild type), **(B)** attenuated C9 mutant, and **(C)** complemented *P. falciparum*. All mice were infected through an intravenous route with 0.3 ml of asynchronous cultures at 1% parasitemia of NF54, C9 mutant (C9-M), and complemented parasite (C9-C) of *P. falciparum*. The PfNSG-IV mice supported the grafting of highly attenuated parasites without needing the adaptation of the parasite to the host.

Three mice for each group were infected with NF54 (Figure 3A), C9-M (Figure 3B), and C9-C (Figure 3C) parasites. Interestingly, all NSG-IV mice showed 100% infection and supported the replication of all *P. falciparum* strains employed (Figure 3). However, we did not see significant (*p* < 0.05) differences in parasite growth in NF54, C9-M, and C9-C parasites in these humanized NSG mice. The short-term human blood chimerism in mouse circulation led to the clearance of NF54 infected erythrocytes on day 13 and 14 post-infection (Figure 3A). However, sufficient numbers of circulating huRBCs allowed C9-M (Figure 3B) and C9-C parasites (Figure 3C) to stay in the periphery until day 22 post-infection. Low standing parasitemia seen with C9-M is attributed to the knock-out of PF3D7_1305500, which may have modulated the host immune response due to the higher secretion of immunoregulatory and anti-inflammatory cytokine, IL-10.

### Humanized Mice Support the Development of Attenuated C9-M *P. falciparum*

**Figure 4:**
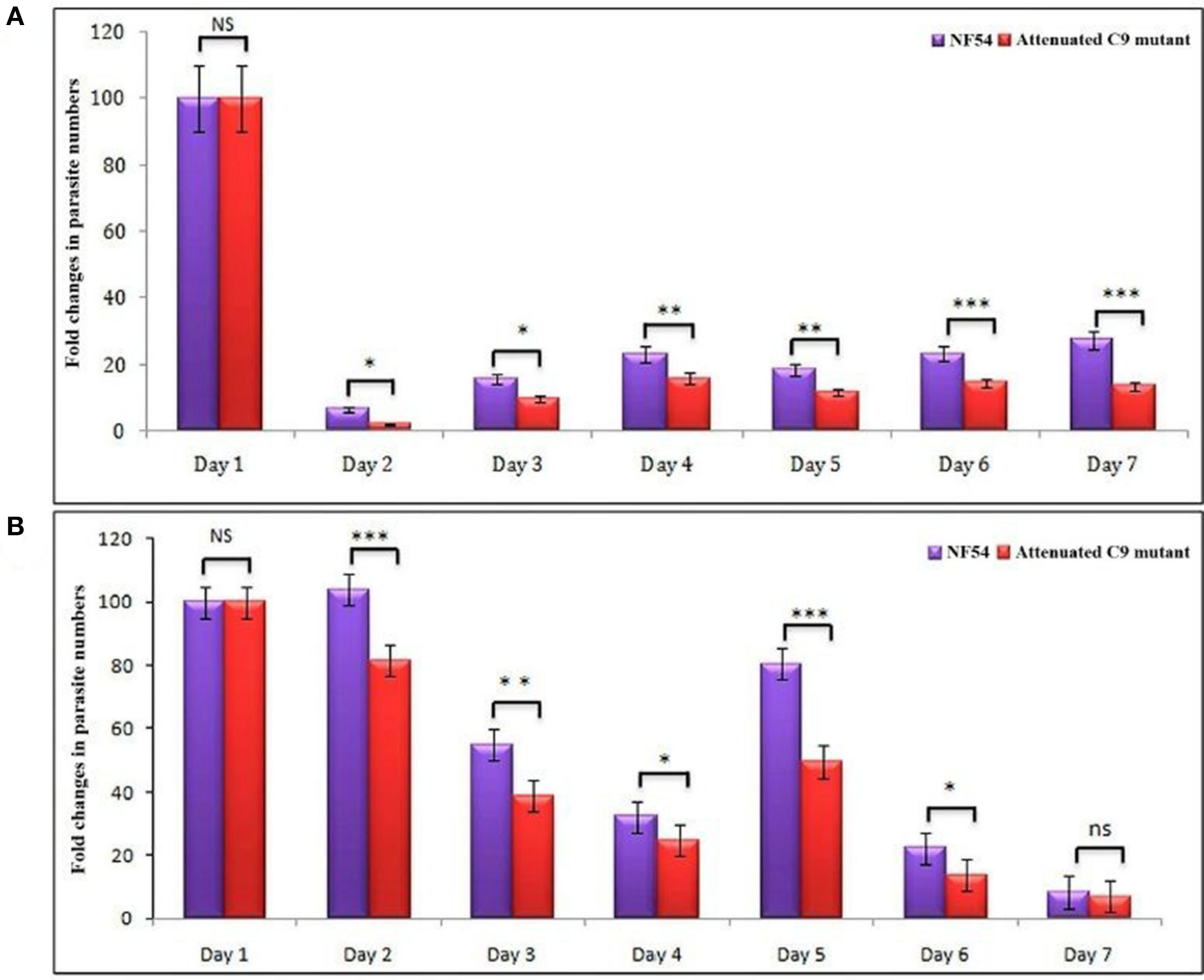
**(A)** *In vitro* and, **(B)** *in vivo* phenotypic characterization of attenuated growth mutants. A bar graph of fold changes in parasite numbers for 7 days of growth revealed a spectrum of attenuated growth phenotypes in C9-M*P. falciparum* parasite until day 6 in comparison to their wild type progenitors. The growth assays were carried-out on the samples collected from *in vitro* cultures and mice infected with wild type and C9-M *P. falciparum*. *p < 0.05; **p < 0.01; ***p < 0.001.

Based on the successful development and replication of different strains of *P. falciparum* (NF54, C9-M, and C9-C) (Figure 3), we next carried out the growth assays to detect the effect of engraftment on the attenuation attributes of mutant parasites. Therefore, a library of unique mutant clones was created from a laboratory line of *P. falciparum* (NF54) using random insertional mutagenesis with a *piggyBac* transposon (33). Fifty percentage attenuation in the *in vitro* culture of intra-erythrocytic C9-M mutant parasites was seen and compared with the wild type (NF54) parasites (Figure 4A). The mutation responsible for attenuation in the growth of C9-M parasite *in vitro* slowed down the parasite growth and replication. As with others (26), the mean number of calculated merozoites remained the same as those seen with the parent parasites (NF54). Interestingly, C9-M parasite did not retain its phenotype when grafted in huRBC-reconstituted NSG mice. Also, we did not see significant differences in the parasitemia pattern of C9-M and wild type (Figures 3A,B) parasites, or in their morphology (Figure 2A). Growth assays were carried out on the blood drawn from the mice harboring C9-M and NF54 parasites (Figure 4B). The growth phenotype (50% growth attenuation) of C9-M parasite in parasite culture and grafted in huRBC-reconstituted humanized mice exhibited a different pattern. The humanized NSG mice supported *P. falciparum* infection and allowed for replication of the C9-M parasite. These attenuated growth mutants survived the residual innate immune responses of the host. Therefore, humanized mice will be useful to study the genotypic and phenotypic characteristics of mutant parasites.

### *In vitro* and *in vivo* Typing of C9-Mutant and C9-Complement Parasite

**Figure 5:**
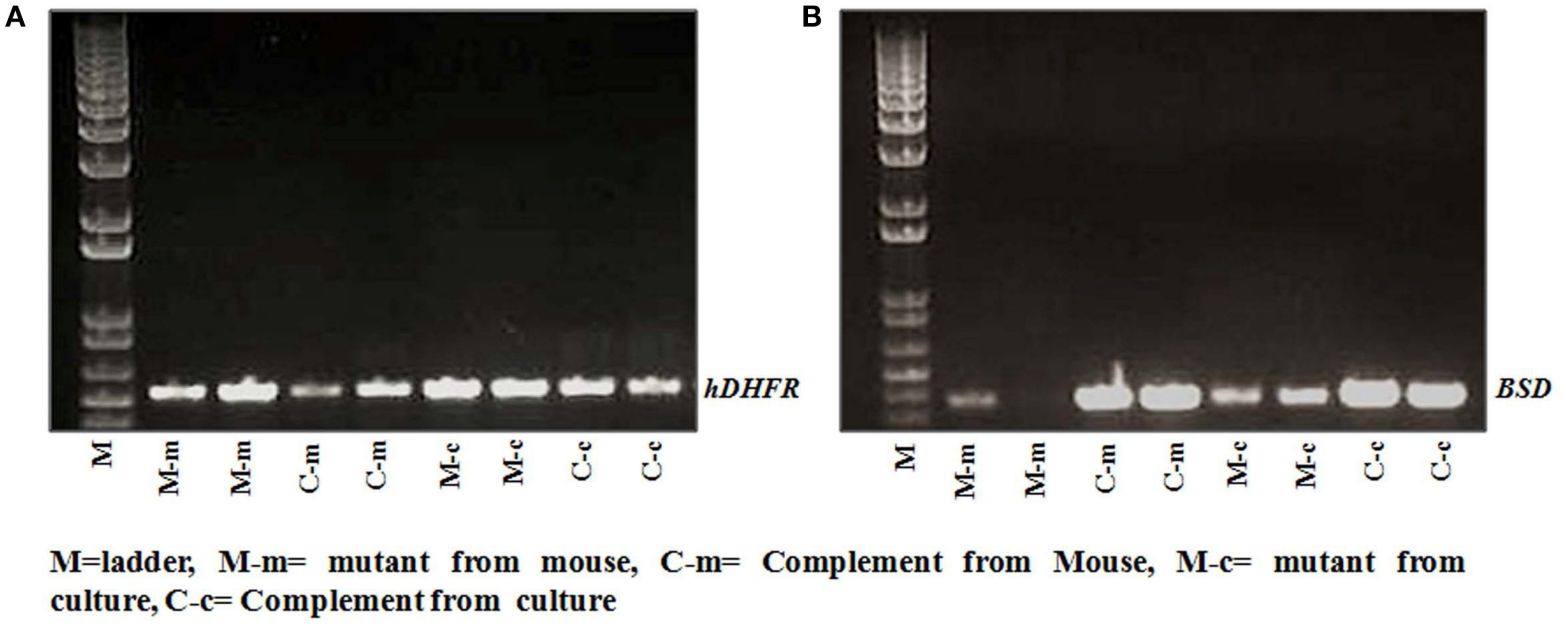
Genotyping of parasite strain(s) used in the study. The presence of **(A)** hDHFR, and **(B)** BSD cassette in integrated parasite genome from cultures, and NSG mice infected with C9-M and C9-C parasites was confirmed in both the strains tested. Lane 2 and 3: C9-M parasites from mouse; lane 4 and 5: C9-C parasite from mouse; lane 6 and 7: C9-M parasite from culture, and lane 8 and 9: C9-C parasite from culture.

We did not see much difference in the growth pattern of wild type and mutant (C9-M) parasite when growth assays were conducted on parasites from culture and grafted humanized mice. Therefore, we decided to characterize both mutant and complement parasite strains. Parasite strains grafted in humanized mice (Pf-huRBC/NSG-IV) were typed for the attenuated C9-M and C9-C phenotype by PCR (Figure 5). Both strains were thawed, cultured, and kept under Blasticidin (BSD) pressure *in vitro* (Figure 5B), and the presence of human-DHFR and BSD selection markers were confirmed with the genomic DNA extracted from C9-M and C9-C parasites from culture and *P. falciparum* infected humanized NSG mice (Figures 5A,B). hDHFR (Figure 5A) and BSD (Figure 5B) showed an amplified product band of 536 and 393 bp of DNA extracted from C9-M and C9-C parasites from culture and mice harboring the parasite, respectively.

### Putative Phosphatase (PF3D7_1305500) Knock-Down Is Correlated With Cytokine Variations in *P. falciparum* Infected Humanized NSG Mice

The idea of the present work is to validate the role of knock-down effect of ribosomal binding protein (PF3D7_1305500) in huRBCs reconstituted humanized mice. Following the confirmation of the development and replication of typed mutant (PF3D7_1305500) parasite in humanized mice, upon injection with 650 μl of huRBC, our results showed the significant (*p* < 0.05) human blood chimerism (proportion of huRBC in mouse's periphery) ranging from 60 to 70% of total erythrocytes. This blood-chimerism was stable over a month and supported rapid and optimal growth of *P. falciparum* (Figures 1, 3) with abundant and very healthy-looking parasites (Figure 2A). Total hematocrit in mouse's periphery showed an average of 55–60% of huRBCs in this protocol (data not shown).

**Figure 6:**
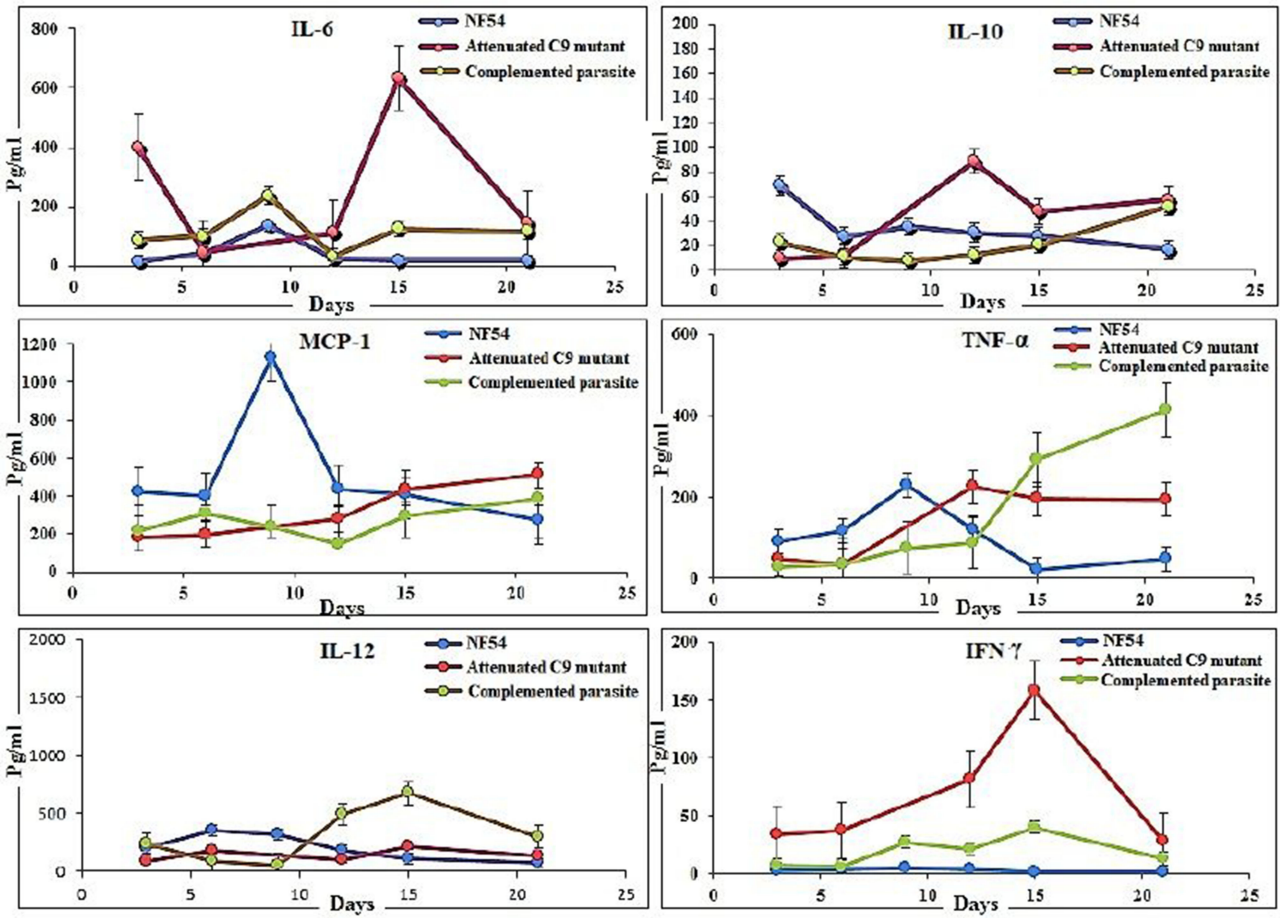
Putative phosphatase (PF3D7_1305500) knock-down is correlated with the reduction in the inflammation in *P. falciparum* infected humanized mice. The determination of cytokines (IL-6, IL-10, MCP-1, TNF-α, IFN-γ, and IL-12) in the serum collected from mice infected with wild type NF54, attenuated C9 mutant, complemented parasites. Results represent the mean of experiment performed using three mice per group, and the standard error was calculated.

Furthermore, the use of a lower dose (450 μl) of huRBC showed an initial establishment of human blood chimerism followed by a decrease, which is most likely due to the inflammation induced by parasites (16). Therefore, we used human RBCs reconstituted NSG-IV mice to analyze inflammatory markers to investigate the role of PF3D7_1305500 RNA binding protein in the intra-erythrocytic development of the parasite. Three mice in each group were reconstituted with huRBCs, given the infectious challenge with NF54, C9-M, and C9-C parasites, and sera were collected at different time-points. The raised levels of IL-6 (632 ± 526Vs 17 ± 8 pg/ml, day 15 post-infection) and IL-10 (88 ± 60 vs. 30 ± 27.41 pg/ml, day 12 post-infection) were quantified in the mice infected with C9-M parasites and compared with NF54 wild type *P. falciparum* (Figure 6). The higher inflammation mounted by the host against the parasite infection is correlated with the clearance of infected and uninfected huRBC from the mouse's periphery. A greater increase in the chemokine MCP-1 (1129 ± 435 vs. 277 ± 16 pg/ml day 9 PI) in NF54 infected mice and the level of IFN-γ (158 ± 73 vs. 1.65 ± 5 pg/ml day 15 PI) in C9-M parasites than NF54 was found to be significant (*p* > 0.05). Interestingly, a greater increase in TNF-α (422 ± 50.63 vs. 49 ± 21.76 pg/ml day, 21 PI) and IL-12 (128 ± 69 vs. 110 ± 56 pg/ml, day 15 PI) was seen in the mice infected with C9-C parasites than those seen with NF54 infected mice (Figure 6). The sub-optimal dose of huRBC grafting resulted in the reduced human blood chimerism and resulting parasitemia. Consistent with other's findings (16), the initial rise of IL6, followed by an increase of IL-12p70 and moderate changes in TNF-α and MCP-1 that are temporally associated with anemia, may play a role in its etiology.

### C9 Mutant *P. falciparum* Partially Escapes the Residual Innate Immunity of Host

**Figure 7:**
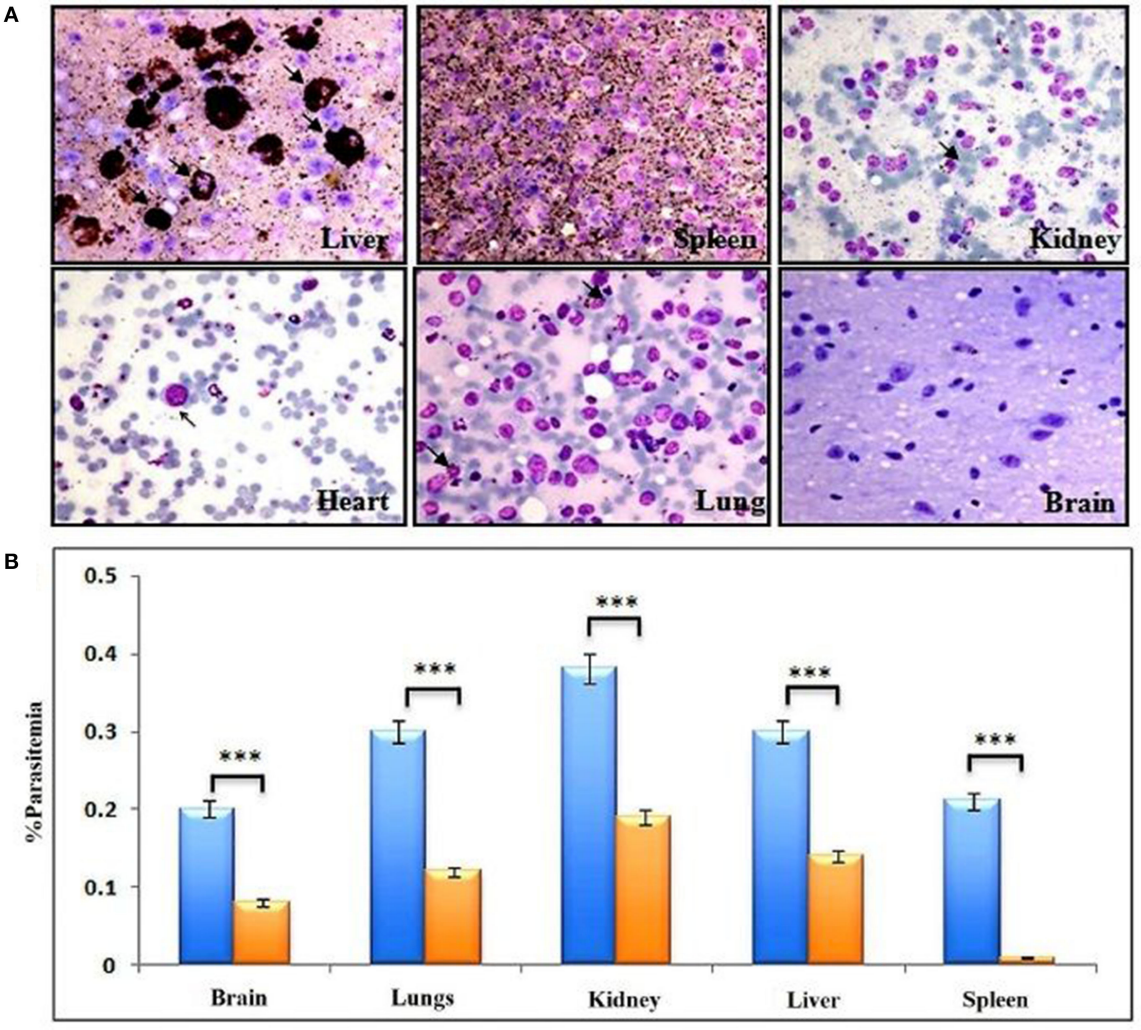
Complete escape from mouse's residual non-adaptive immunity is not possible by C9-M parasite. **(A)** Representative Giemsa staining of smears drawn from various organs 11 days post-infection from three different NSG mice (upper panels: liver, spleen, and kidney, lower panels: heart, lungs, and brain). Arrows indicate C9-M parasitized huRBCs. The presence of parasites in different organs, their phagocytosis, and subsequent release of pigment from active phagocytes, mainly macrophages, is an example of active residual innate immune effectors, **(B)** parasite count in different organs, blue bars; C9-M, red bars; C9-C parasites. ***p < 0.001.

Cells of monocyte-macrophage lineage are continuously recruited and play an important role in the clearance of the parasite from mouse circulation, but mutant parasites reside for long periods of time. Therefore, we next decided to confirm the importance of monocyte/macrophage in the clearance of parasites. The macrophages are the main subsets recruited all over the course of infection, and found to be more active than other phagocytes at ingesting infected and uninfected huRBCs in the peritoneum (9, 34–36) (Supplementary Figure 1). C9-M parasite showed 100% infectivity and slow growth upon being grafted in humanized NSG mice, and parasitemia was seen to decrease by day 12 post-infection (Figure 3B). These mutant parasites were found sequestered in the liver, kidney, and brain to survive the host's residual innate immune responses (Figures 7A,B). The phagocytosis of uninfected and infected huRBCs and subsequent release of pigment suggested that complete escape of parasite from host's non-adaptive immune response was not possible (Figure 7A). Furthermore, our results suggest that the C9-M parasite may have employed evasion mechanism(s) to survive the residual innate immune response of the host. Our findings describe the role of macrophages (MP) in the rejection of both uninfected and infected huRBC by two mechanisms: the release of inflammatory mediators for the activation of monocytes, that then result in increased erythro-phagocytosis. The role of macrophages in xenograft rejection is shown by the infiltration of leukocytes during the rejection of pig-to-primate xenografts (37, 38). Furthermore, selective macrophage depletion in immunocompetent rodents showed significant delays in cellular infiltration and xenograft rejection (39, 40).

### NSG-IV Mice Supported the C9-M *P. falciparum* for the Development of Gametocytes (Propagation Carriers)

The propagation of the parasite from one host to another is important to confirm the replication of the parasite. NF54-wild type strain is known to develop gametocyte *in vitro* culture, which was supported by the humanized mice to induce the production of gametocytes (Figure 2B, lower panel, 1st row; d3, d6, d9). In addition to the remarkable growth and development of C9-M parasites seen in NSG-IV mice, gametocytes were developed in humanized mice (Figure 2B, lower panel, 2nd row; d3, d6, d9). These sexual forms (Figure 2B, lower panel) were frequently seen up to stage V. The observation of stage V gametocytes implies that infected huRBC are able to survive in mouse's circulation for at least 9 days without suffering any immune-mediated insult when NSG-IV protocol was used. We tried to feed mosquitoes on the developed gametocytes to demonstrate their infectivity, but failed to see the development of oocyte on day 11 followed by the development of sporozoites on day 16 or 17. We think more efforts are needed to demonstrate the infectivity of gametocytes developed in Pf-NSG-IV mice.

### huRBC Reconstituted TK/NOG Mouse: A Better Strain for Humanization

**Figure 8:**
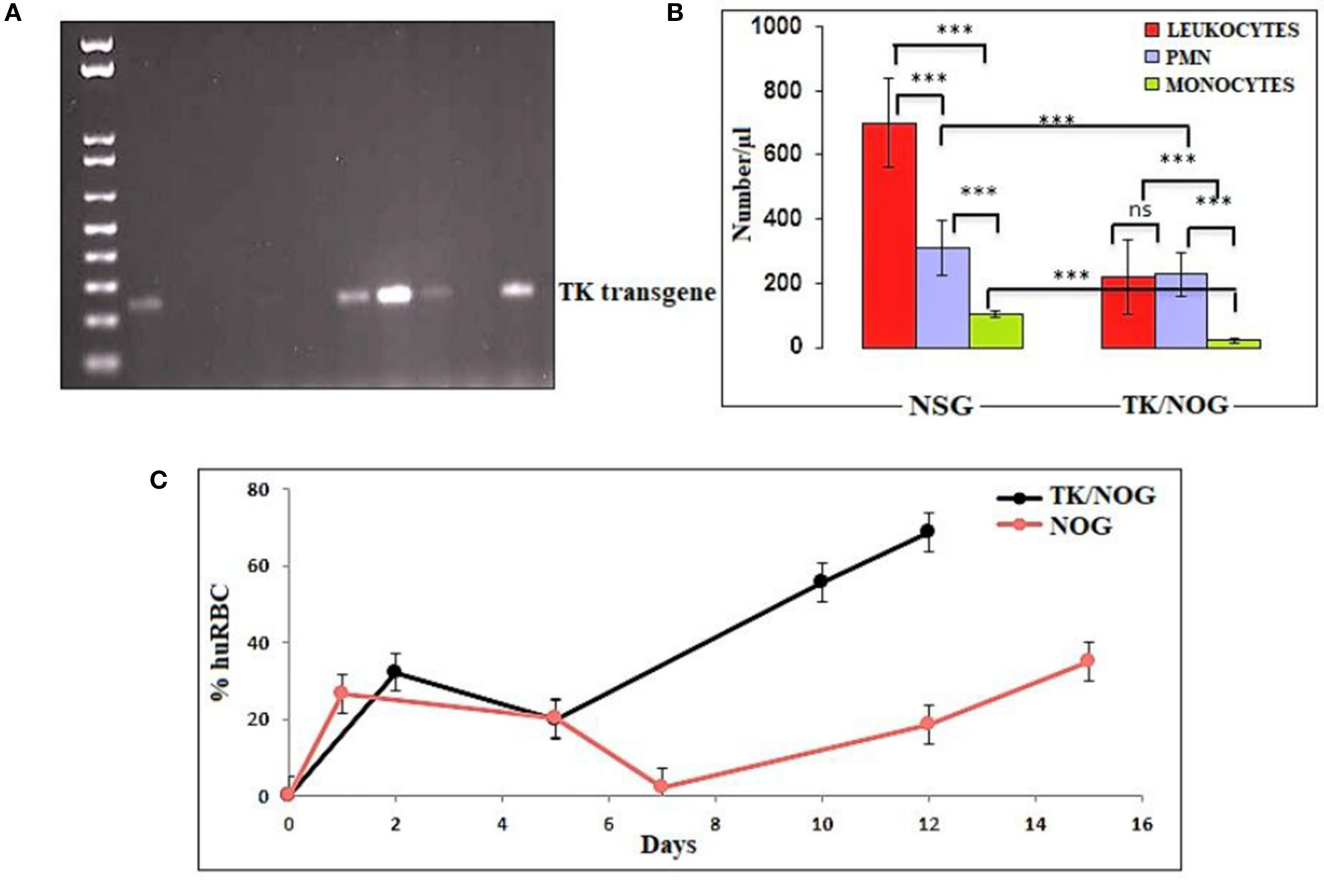
Thymidine kinase transgene expression and enhanced huRBC grafting. **(A)** All litters were genotyped with gDNA samples extracted from tail snips by PCR, and amplified transgene DNA (Thymidine Kinase, 236 bp) was visualized at 1% agarose, **(B)** The reduction in the number of innate immune cells of TK/NOG and NSG mice was compared **(C)** the greater human blood chimerization (huRBC grafting) is seen in TK/NOG mice than NSG mice. ***p < 0.001.

**Figure 9:**
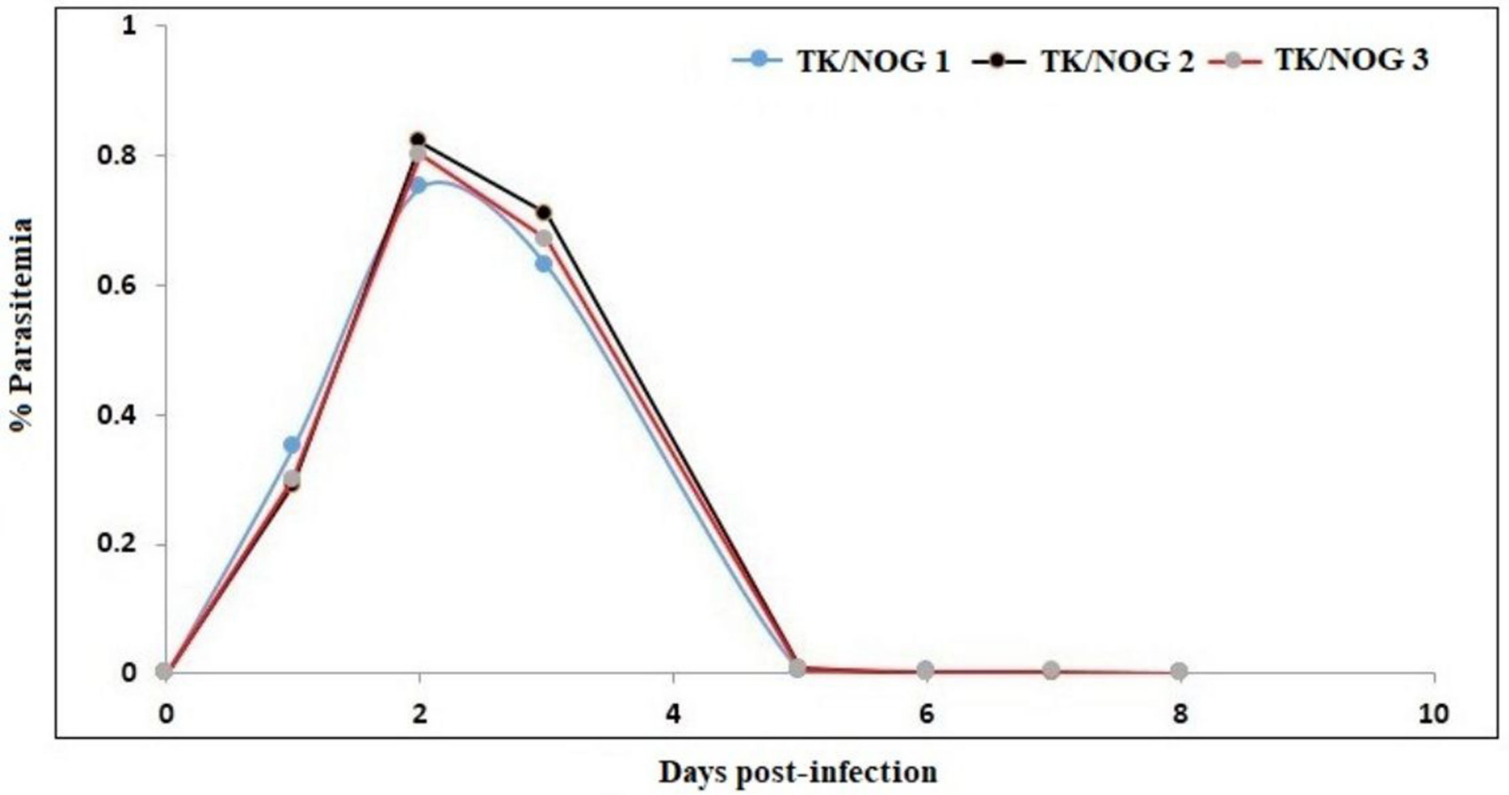
Determination of the half-life of huRBC in mouse's circulation. Mice were intravenously inoculated with 0.3 ml of asynchronous cultures at 1% parasitemia of NF54 strain of *P. falciparum* on day 0. The parasitemia in mice was expressed as the percentage of *P. falciparum-*huRBC in the total RBC observed on thin smears. The data shown is from the first day of detectable parasitemia up to the day the mice had huRBCs in their circulation.

We wanted to study the transgenic strain (32, 41–54) (expressing the thymidine kinase transgene on mouse's hepatocyte) primarily dedicated for developing the human liver-chimeric mice to examine the inflammatory disorders and liver metabolism of drugs. Since this strain is of NOG background, we thought it could support the reconstitution of huRBCs and *P. falciparum* infection. Thus, this mouse strain was deployed in the present study, set to breed, and littermates were genotyped by the established PCR method (32). DNA transgene showed an amplified band of thymidine kinase (Figure 8A) on the agarose gel. The absolute numbers of innate immune effectors (Leukocytes, monocytes, and polymorphonuclears) in TK/NOG were observed to be lesser than in NSG mice (Figure 8B). Next, we wanted to assess the importance of thymidine kinase transgene expression in TK/NOG mice. Therefore, TK/NOG and NSG mice were treated with clodronate-loaded liposomes to check the production of excessively recruited monocytes/macrophages, and animals were intravenously reconstituted with huRBCs. TK/NOG mice supported the significant and long-term huRBC chimerization as compared to NSG mice (Figure 8C). We went on to estimate the half-life (4–5 days) of huRBC to determine the survival of huRBC in mouse periphery (Figure 9). The human blood chimerism was found better (75%) in TK/NOG mice than NSG mice (15–20%) by day 12 post-huRBCs injections (Figure 8C).

**Figure 10:**
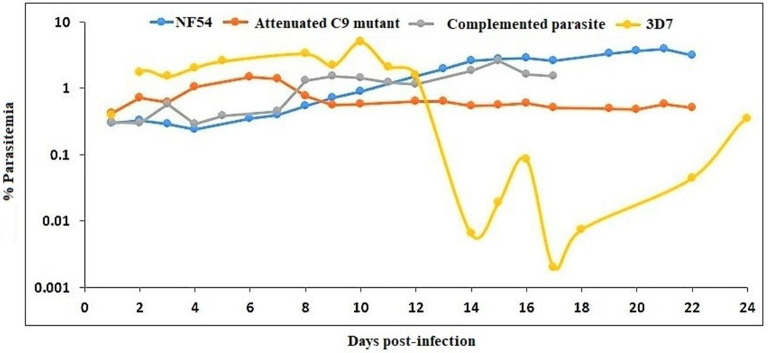
NF54 (wild type), attenuated C9-M, complemented C9-C, and 3D7 parasite strains are supported by TK/NOG mice. The trend of parasitemia is the mean of three mice each of all parasite strains tested. Mice were intravenously inoculated with 0.3 ml of asynchronous cultures at 1% *P. falciparum* parasitemia on day 0. Mice were supplied with huRBC every 3 days. Parasitemia in the mice was expressed as the percentage of *P. falciparum* parasitized huRBCs in total RBC counted on thin blood smears. The presented data is from the first day of detectable parasitemia.

huRBC reconstituted TK/NOG received infection with parental NF54, C9-M, C9-C, and 3D7 (three mice each) strain of *P. falciparum* (Figure 10). All mice receiving infection were seen parasitized by day 1 post-inoculation using IV protocol.

The stable blood-chimerism was seen with huRBs reconstituted TK/NOG (Figure 8C). These mice supported rapid and optimal growth of all *P. falciparum* strains tested (Figure 10). Except for 3D7, all strains (NF54, C9-M, C9-C) showed consistency in parasitemia pattern. Lack in the adaptation of the parasite to the host was attributed to variations seen in the pattern of 3D7 parasitemia. The 3D7 strain made attempts to accommodate with the host's immune system in TK/NOG mice for nearly 2 weeks, and adapted parasites exhibited normal growth and development of 3D7 by day 17 post-infection (Figure 10).

## Discussion

**Figure 11:**
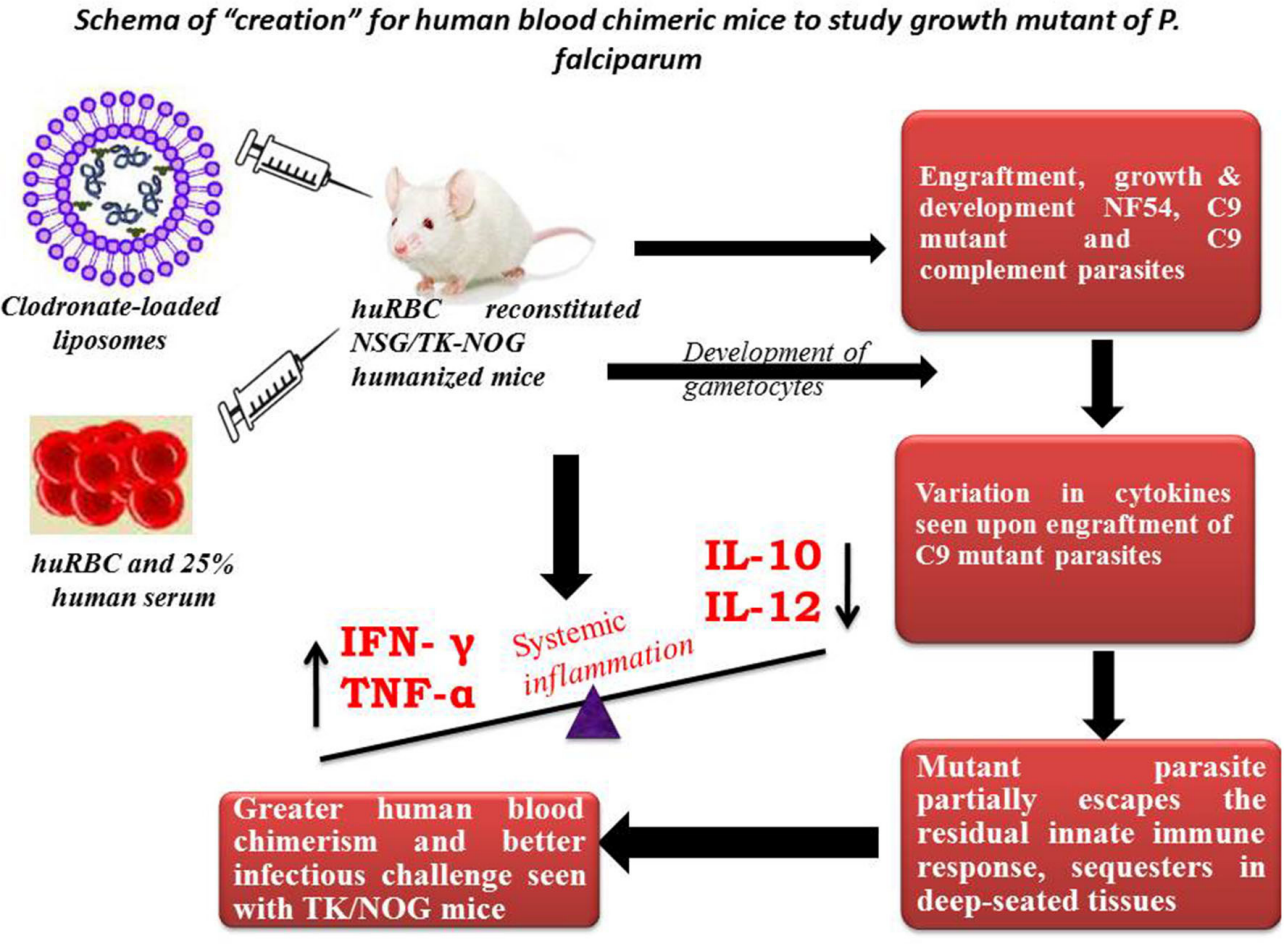
Schema of “creation” for human blood chimeric mice to study the growth mutant of *P. falciparum*. huRBCs reconstituted NSG mice showing variation in the cytokine levels of C9-mutant and C9-complement parasites as compared to wild type NF54. This model indicates the escape-mechanism-like phenomenon since mutant parasites reside for longer in the deep-seated tissues and evade the residual innate immunity of the host.

This study is part of our efforts to develop understanding on experimental mouse model(s) to study unknown asexual blood stage genes/RNA binding proteins of *P. falciparum*. As with others (25), our *in vitro* study showed that attenuated C9-M did not lead the normal cell cycle due to knock-down of PF3D7_1305500. This study suggests the importance of this atypical phosphatase in the regulation of the *P. falciparum* cell cycle. The attenuated C9 knock-out created by random insertional mutagenesis was used in this study to attest the *in vitro* studies (26). Moreover, the importance of PF3D7_1305500 playing a crucial role in the growth and development of asexual blood stage *P. falciparum* was confirmed in humanized mice (Figure 11).

We succeeded to implement a reproducible infection by using different *P. falciparum* strains in a PfhuRBC-NSG-IV humanized mouse (16) to understand the development and replication of attenuated C9-M parasites. Consistent with the previously published findings (16), we confirm that employing an intravenous route for both parasite and huRBC delivery, clodronate-loaded liposomal suspension for macrophage suppression, and the IL-2R-γ mutation on NOD/SCID genetic background, helped in achieving a model with greater reproducibility which supported the infection with different strains, including the severely attenuated C9-M.

The present study shows that *P. falciparum* huRBC/NSG-IV model (huRBC-reconstituted NSG mice infected with NF54, C9-M, and C9-C parasites) may be useful to study the immune responses evoked against the grafted parasite, and possible survival mechanisms employed by the parasites. The additional defects in the innate immune system of NSG mice are most likely related to the defective activation of phagocytes which led to the reduced *P. falciparum* selection pressure (11).

Our results show that: (1) *P. falciparum* induces strain dependent moderate inflammation characterized by the release of inflammatory cytokines in the serum; (2) evolution of parasitemia in mouse's periphery remained stable but short-term blood chimerization (insufficient huRBCs) in the circulation leads to the clearance of parasites driven by the pro-inflammatory cytokines; (3) growth and development of all *P. falciparum* strains was supported by the developed humanized mice, indicating that, in contrast to Aotus, Saimiri, and previous mouse models, there is no requirement for the preliminary adaptation of the parasite to PfhuRBC-NSG-IV host. The slower growth of C9-M indicated that the parasite could retain the mutant phenotype upon being grafted in human RBCs reconstituted humanized mice. However, there was no difference seen in the parasitemia patterns of grafted parental NF54, C9-M, and C9-C parasites. This shows the relevance of this mouse as it helps in understanding the behavior and relevance of C9 mutation in the growth and replication of the asexual blood stage infection of *P. falciparum* without tampering its genotype and phenotype; (4) migration of parasites from the mouse's periphery to deep-seated organs with the extended residence indicated that C9-M parasites may have employed molecular mechanism(s) to partially evade the host's innate immune response; (5) humanized mouse supported the development of the sexual stage of *P. falciparum*. Stage III, IV, and V gametocytes were seen in the peripheral blood but their infectivity could not be demonstrated; and (6) transgenic/immunodeficient (TK/NOG) mice were shown to better control the non-adaptive immune response as compared to NSG mice, and therefore higher human blood chimerism was seen when reconstituted with huRBCs in the mouse prepared by clo-lip treatment. huRBCs grafted TK/NOG mice showed greater susceptibility toward infection with stable parasitemia in all *P. falciparum* strains tested (Figure 10).

Results obtained with the humanized mouse model, as reported earlier (9, 13, 16), concur to suggest that the model is closer to events recorded in humans (for instance, the receptivity shown toward non-adapted parasites and gametocyte production).

The disruption of any biochemical process affects the normal pattern of metabolic events which leads to the completion of the asexual blood stage development of *P. falciparum*. The *in vitro* findings (26) showed the significance of PF3D7_1305500 (C9-M) in the regulation of the asexual blood stage cell cycle of *P. falciparum*. The delayed transition of pre-S trophozoite to S/M schizonts showed the checkpoint of the parasite growth cycle. The rescue of C9-M phenotype by the genetic complementation confirmed the requirement of PF3D7_1305500 for normal growth and development of *P. falciparum in vitro* (26). Further, the *in vivo* selection process led to the variants of *P. falciparum* carrying potential rearrangements to variable molecules encoding antigenic determinants (55). The engraftment of C9-M in humanized mice shows the elicitation of active residual innate immune responses. The detailed analysis better defined the role of each cytokines/chemokines produced against the grafted C9-M and C9-C *P. falciparum*. And, our findings contribute to understanding the delicate balance between inflammation control and *P. falciparum* survival in humanized mice (Supplementary Figure 1).

The relationship between the knock-out effect of putative phosphatase and the mouse's ability to both tolerate the graft and produce fewer inflammatory mediators is an interesting finding. A marginal increase in the inflammatory mediator (IL-6) and immune-suppressor (IL-10) than their wild type progenitors was seen. We believe that improved survival of infected and uninfected huRBCs is associated with a decrease in the inflammatory cytokine and a slight increase in the production of IL-10 by the cells of myeloid origin in C9-M harboring mice. Therefore, controlled pro-inflammatory cytokines allowing the sustained parasite development in deep-seated organs indicate that *P. falciparum* might be employing the escape mechanisms to survive the host's residual innate immunity (55). We measured increased levels of TNF-α and IL-12 in C9-M and C9-C parasites in infected humanized mice. In addition, we saw an increase in serum levels of MCP-1 in NF54 infected humanized mice. These results are in agreement with earlier findings (16). The increase of IL-12p70, TNF-α, and MCP-1 are temporally associated with anemia which might play a role in its etiology and inflammation. Lastly, we introduced a very promising transgenic/immunodeficient strain (32, 41–45, 47–51, 56, 57), primarily dedicated for developing human liver chimeric mice to study human liver physiology, drug metabolism, and liver pathogenesis of viral etiology or liver regeneration. However, we reconstituted TK/NOG mice with huRBC and saw better blood chimerism to allow *P. falciparum* replication. The better control of residual non-adaptive immune effectors such as leukocytes, PMNs, and monocytes/macrophages (49, 58) also contributed to the parasite's growth and development. The monocytes/macrophage bears their most critical function in the clearance of parasitized or un-parasitized huRBCs from mouse's periphery (9). The significant human blood chimerization supported by the TK/NOG mice when grafted with huRBC advocates for better replication of *P. falciparum* (49).

## Conclusion and Future Perspectives

The emergence of resistance to frontline drugs and the lack of diverse therapeutic agents necessitates the finding of effective anti-malarial drugs and the identification of new drug targets (7, 59–65). Our *P. falciparum*-NSG-IV model may allow the study of asexual blood stage growth mutant(s) and their effect on the human system. This mouse shows reproducibility of both huRBC grafting and parasite survival, less day to day variation in parasitemia, does not require preliminary adaptation of parasite strains to the mouse, and supports the development of even attenuated *P. falciparum*. The evasion mechanisms employed by the parasites help survive against the host's residual immune responses and raises the possibility of partial sequestration of parasites in deep-seated organs, which are key findings of the present work. The delineation of *in vivo* function and behavior of attenuated C9-M parasites gene (PF3D7_130550) in a humanized mouse might give insights into this unknown protein, important for the growth and development of the asexual blood stage of *P. falciparum*.

## Data Availability Statement

The raw data supporting the conclusions of this article will be made available by the authors, without undue reservation.

## Ethics Statement

The animal study was reviewed and approved by The Second Hospital of Jilin University, Changchun, China.

## Author Contributions

LZ and MT conceived and designed the experiments, analyzed the data, and wrote the paper. LZ, J-LL, M-XJ, DT, L-YW, and CC performed the experiments and contributed materials/analysis tools. All authors contributed to the article and approved the submitted version.

## Conflict of Interest

The authors declare that the research was conducted in the absence of any commercial or financial relationships that could be construed as a potential conflict of interest.

## Supplementary Material

Supplementary Figure 1 **(a)** HuRBCs are massively engulfed by macrophages in the peritoneum of mice administered with human RBCs. **(b)** Sizable rafting of huRBCs was seen when co-injected with clodronate loaded liposomes (9). Black Square-HuRBC, Plain circle- HuRBC+ Clo-lip, Open circle- HuRBC+ NIMP.

## Abbreviations

ORF: open reading frame
Clo-lip: Clodronate-loaded liposomes
NSG: NOD/SCID/IL-2rg^−/^
huRBC: Human Red Blood Cells
TK/NOG: Thymidine kinase-NOD/SCID/IL-2rg^−^/
C9 mutant: PF3D7_1305500
MCV: mean corpuscular volume
hDHFR: human dihydrofolate reeducates
BSD: Blasticidin.
