# Supplementary figures and images for: Attenuated P. falciparum Parasite Shows Cytokine Variations in Humanized Mice
Source: Front Immunol. 2020 Sep 11;11:1801. doi: 10.3389/fimmu.2020.01801 (PMC7516016; doi:10.3389/fimmu.2020.01801)

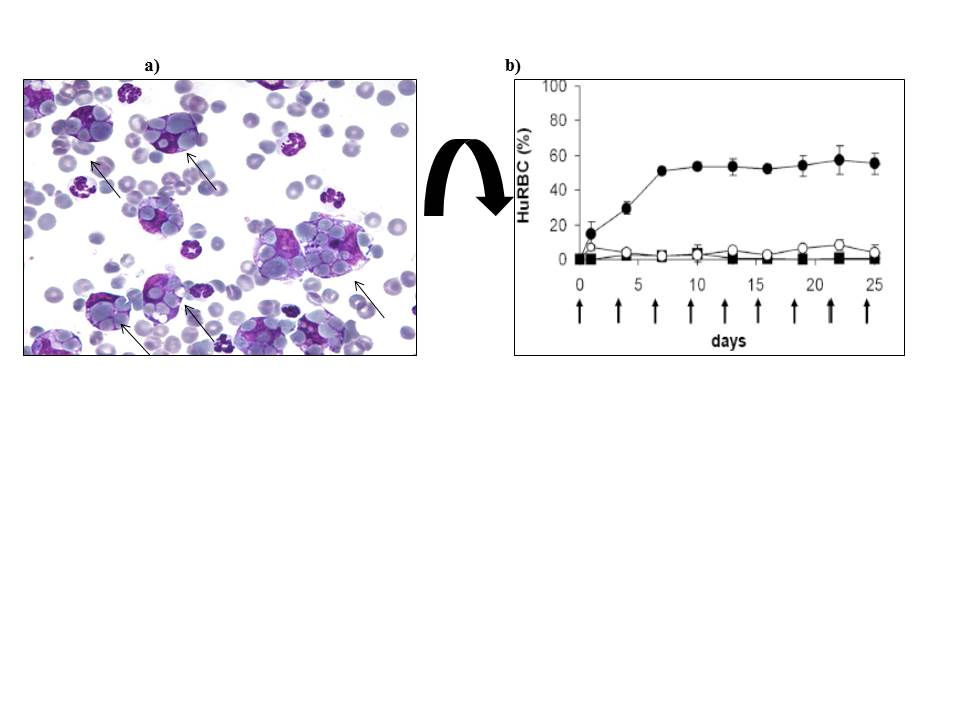

Supplement: Supplementary Figure 1 — (a) HuRBCs are massively engulfed by macrophages in the peritoneum of mice administered with human RBCs. (b) Sizable rafting of huRBCs was seen when co-injected with clodronate loaded liposomes (9). Black Square-HuRBC, Plain circle- HuRBC+ Clo-lip, Open circle- HuRBC+ NIMP. [file Image_1.jpeg]
